# Compassion: Learning Needs and Training Opportunities—a Survey Among Palliative Healthcare Providers in Italy

**DOI:** 10.1007/s13187-021-02093-0

**Published:** 2022-03-04

**Authors:** Andrea Bovero, Beatrice Adriano, Irene Di Girolamo, Chiara Tosi, Luciano Orsi, Cinzia Ricetto, Rossana Botto

**Affiliations:** 1 Department of Neuroscience, Clinical Psychology Unit, University of Turin, “Città Della Salute E Della Scienza” Hospital, Corso Bramante 88, 10126 Turin, Italy; 2 Italian Society of Palliative Care (SICP), Milan, Italy; 3 Italian Society of Palliative Care (SICP), Piedmont-Aosta Valley, Italy

**Keywords:** Psychological care, Education and training, End-of-life care, Terminal care

## Abstract

Compassion is a key quality in palliative care; however, there is a lack of evidence of the need to discuss the theme of compassion and professionals’ training in the subject. The study aimed to investigate the knowledge of the construct of a sample of Italian healthcare professionals (HCPs) working in palliative care. In addition, their learning needs and training opportunities were explored. An online survey was completed by 330 HCPs. It was divided into five sections which examined knowledge of the construct of compassion and the perception of its utility in palliative care, the activities carried out in eventual training in compassion, and professionals’ learning needs thereof. Professionals who had knowledge of the right definition of compassion considered it more useful and training more necessary. Most of the sample never received training about compassion. However, 97% of those who received training believed it to be necessary. Satisfaction with training was higher among those who received multidisciplinary team education. Training occasions are relatively rare in the Italian context, although they seem to increase knowledge and awareness about the construct utility and training necessity. Besides, multidisciplinary team training seems to be more satisfying. Offering team training on compassion can promote a deeper awareness of it and of its utility in clinical practice.

## Introduction

Compassion is a central issue in the daily assistance to patients at the end of life. Etymologically, the term “compassion,” which means “suffering together with another,” derives from the Latin “*com*” (together with) and *pati* (to suffer) [1]. It is a key quality through which healthcare providers emotionally perceive patients’ suffering and needs by wanting to alleviate it with relational understanding and actions [2].

Compassion is often confused with the related constructs of empathy and pity, but they refer to different meanings [3]. Empathy is an aptitude, the emotional and cognitive ability to recognize and “understand an individual’s suffering through emotional resonance” [4]; it does not require prosocial motivation and behavior, which are basic attributes of compassion (*ibidem*). Pity is the feeling of painful and caring participation in the suffering of someone considered inferior and weak [3]. Not confusing the constructs allows more focused clinical interventions and permits the healthcare professionals to better manage their care activities. It is important to emphasize that knowing the definition is different from understanding or providing it; however, this is a preliminary survey. Knowledge is necessary and preparatory to compassion implementation and training. Moreover, compassion is not a simply innate trait embedded in people’s character; it is also a quality that can be taught and trained [5].

Despite the growing literature about this topic, it is not so clear how much compassion is known in the palliative care field, and what are the learning needs and training opportunities, especially in the Italian context. To our knowledge, in Italy, there is no research about compassion in palliative care; however, the importance of this topic is irrefutable. Compassion can improve the quality of life of the patient and can allow greater satisfaction of the family and a better healthcare system [3].

Compassion skills can reduce rates of burnout and distress of healthcare workers [6].

Because of the lack of literature, and the requests for learning that we have recently received from HCPs through clinical activities, and based on Sinclair’s distinction among pity, empathy, and compassion, this survey aimed to investigate the knowledge of the compassion construct in a sample of HCPs working in palliative care. Moreover, in the Italian scenario, there are no best practices or clearly defined guidelines to provide adequate and effective compassionate care training in palliative care. For these reasons, we also assessed the HC workers’ training opportunities and professionals’ learning needs on compassion.

## Methods

### Study Population

The target population was the Italian healthcare providers working in palliative care and the sample consisted of 330 palliative healthcare providers recruited between January and April 2020. Participants from all over Italy completed a Google Form survey created ad hoc for the research. The Google Form link was available online on the Italian Palliative Care Society (SICP) website. We used SICP website for the recruitment because SICP is the only existing scientific Italian palliative care society, and approximately a thousand palliative care workers belonging to different professional categories are SICP members. This let us reach participants and reduced population selection biases. The link was also sent through SICP’s newsletter to subscribers to the SICP website. Finally, the same link was sent to 220 Italian palliative care health providers and, in turn, they were asked to send it to other palliative care professionals. Data were collected through a snowball sampling. The Google Form link remained active for 2 months. The only eligibility criterion required was being a palliative care health provider. The online survey included an introduction section encompassing information about the time required to complete the questionnaire, voluntary participation, and the aim of the study.

Participants gave their informed consent and completed the survey anonymously.

### Data Collection

The survey was composed of 15 multiple-choice questions divided into 5 sections. It was created after a review of previous literature carried out to define the construct of compassion and identify different types of compassion training.

The first section collected socio-demographic characteristics including gender, age, years of work in healthcare and in palliative care, professional role, geographic area of work, and care setting.

The second section examined knowledge of the construct of compassion and the perception of its utility in the palliative clinical practice. More in detail, participants were asked to select the right definition of compassion, choosing from three different options: the definition of a pity-based response, the definition of empathy, and the definition of compassion as described by Sinclair et al. [7].

The next two sections were addressed to the analysis of the modalities and the activities carried out in eventual education about compassion, with a specific in-depth analysis of team training. Participants were asked whether they had received education or training about compassion (if yes, how much they were satisfied) and whether it is useful for clinical practice in palliative care. Then, the survey investigated the learning contexts where education on compassion was received (participants could choose among academic studies, conferences or congress, Masters, compassion training courses, team training, and other) and the learning strategies used (supervision, focus group, frontal lesson, experiential group activities, and other). Finally, the professional profiles of the trainers for the education on compassion to the palliative care teams were investigated: participants could answer choosing among psychologist/psychotherapist, philosopher, counselor, physician, nurse, and other.

The final portion of the survey involved the perceived need to discuss and train on the theme of compassion in palliative care.

All the questions are listed in the tables below.

### Statistical Analyses

Data administration and statistical analysis were performed using SPSS software, Version 24. Descriptive statistics, such as frequencies, means, and standard deviations, were used to describe the sample.

Associations between variables were investigated using *χ*^2^ tests, *t* tests, analysis of variance, Kruskal–Wallis, and *U* of Mann–Whitney tests. The Bonferroni post hoc test was used to identify which groups showed significant differences. *ρ* values ≤ 0.05 were considered statistically significant.

## Results

### Socio-demographic Data of the Sample

Socio-demographic data are shown in Table 1.

**Table 1:** Socio-demographic data of the sample

Socio-demographic data	*N* = 330	%
Gender		
Men Women	103 227	31.2 68.8
Age		
20–30 30–40 40–50 50–60 60–70 ˃ 70	32 43 103 105 44 3	9.7 13.1 31.2 31.8 13.3 0.9
Years of working experience		
0–2 2–5 5–10 10–20 ˃ 20	25 25 27 95 158	7.7 7.7 8.1 28.7 47.8
Years worked in palliative care		
0–2 2–5 5–10 10–20 ˃ 20	65 64 61 107 33	19.8 19.4 18.5 32.4 9.9
Workplace		
Hospital Hospice Home Care Unit Nursing Home	77 103 115 35	23.2 31.3 34.9 10.6
Profession		
Physician Psychologist Nurse Healthcare assistant Spiritual assistant Physiotherapist Occupational therapist Other	130 60 103 8 8 6 1 14	39.4 18.2 31.2 2.4 2.4 1.8 0.3 4.3

### Frequencies of the Answers to the Survey Questions

Frequencies distributions are shown in Table 2.

**Table 2:** Frequency of answers to the survey questions

What does compassion mean? *N* = 330		
	***N***	**%**
Compassion	234	70.9
Empathy	66	20.0
Pity-based response	28	8.5
“I don’t know”	2	0.6
How much do you think compassion is useful for palliative care clinical practice? *N* = 330		
	***N***	**%**
Not useful at all	0	0
Not very useful	7	2.1
Quite useful	86	26.1
Very useful	237	71.8
Have you ever received education or training on compassion? *N* = 330		
	***N***	**%**
Yes	97	29.4
No	233	70.6
In how many learning contexts did you receive compassion training? *N* = 97		
	***N***	**%**
1 context	41	42.3
2 contexts	39	40.2
3 or more contexts	17	17.5
In which context did you receive training? *N* = 97		
	N*	**%**
Team Education	63	64.9
Conventions/Congresses	48	49.5
Master’s Degrees	27	27.8
Specific Courses relating to the Subject	25	25.8
Academical Studies	11	11.3
Other	6	6.2
If you received training in a palliative care team setting, which professional figure was responsible for compassion education? *N* = 63		
	N**	**%**
Psychologist	45	71.4
Physician	19	30.2
Nurse	8	12.7
Philosopher	5	7.9
Counsellor	5	7.9
Thanatologist	2	3.2
Other (Frank Ostaseski)	2	3.2
Spiritual assistant	1	1.6
If you received training in a palliative care team setting, which learning strategies did you use during compassion education? *N* = 63		
	N***	**%**
Experiential group’s activities	33	52.4
Lectures	24	38.1
Team supervisions	15	23.8
Focus Groups	14	8.8
Other	2	3.2
If you received training on compassion, how much are you satisfied with it? *N* = 97		
	***N***	**%**
Not satisfied at all	1	1.0
Not very satisfied	15	15.5
Quite satisfied	58	59.8
Very satisfied	23	23.7
How much do you think educational programs and training opportunities on compassion are necessary? *N* = 330		
	***N***	**%**
Not necessary at all	4	1.2
Not very necessary	6	1.8
Quite necessary	161	48.8
Very necessary	159	48.2

*N might be >97 because participants could receive training in more than one learning context

**N might be >63 because participants could simultaneously receive training from more than one professional figure

***N might be >63 because participants could receive team training through more than one learning strategy

### Associations between Survey Answers

Participants who knew the right meaning of compassion believed it to be a more useful construct (mean = 3.75, sd = 0.454) and thought that training programs were more necessary (mean = 3.5, sd = 0.58) than those who did not know the right definition of compassion (mean = 3.59, sd = 0.576; mean = 3.32, sd = 0.55) (*t*=$-$ 2.451, *p* ≤ 0.01; *t*=$-$ 2.609, *ρ* ≤ 0.01).

Participants who had received training or education about compassion (only 29.4% of the sample) considered it to be more useful (*m* = 3.81, sd = 0.391) and training more necessary (*m* = 3.56, sd = 0.5) in palliative care clinical practice compared to those who had never received training (*m* = 3.65, sd = 0.521; *m* = 3.41, sd = 0.6) (*t*=$-$ 3.046, *ρ* < 0.01; *p* ≤ 0.05).

Utility of compassion in palliative care positively correlated with the “perceived necessity of training in it” (*r* = 0.395, *p* ≤ 0.01).

There was a positive correlation between satisfaction with compassion education and the number of experienced learning contexts (*r* = 0.412, *p* ≤ 0.01).

Perceived satisfaction with the received training was significantly higher among those who had received it as a team education (mean = 3.22, sd = 0.629) compared to those who did not receive it (mean = 2.76, sd = 0.614) (*t*= $-$ 3.448, *ρ* ≤ 0.01).

In the present study, only the results of the most impactful analyses have been reported. No significant results were found by analyzing by age, years of experience, occupation, and workplace.

## Discussion

Seventy percent of the sample selected the correct meaning of compassion among various options, even if only 30% had received specific compassion training. This might underline how compassion could be an innate feature.

Nevertheless, training on compassion were considered by most of participants as very useful and necessary in end-of life clinical practice, also considering the importance they attributed to compassionate care.

Moreover, professionals who had a better knowledge of the construct expressed a higher perceived utility of compassion in palliative care practice and a higher necessity of training. Finally, utility of compassion and perceived necessity of training correlated positively. This could indicate a sort of virtuous circle according to which the more training is received, the more knowledge of compassion is enhanced, and the more importance is given to training and compassionate care itself. Results showed more satisfaction with training among those who received team-based training or education and enhanced satisfaction when professionals were given access to more than one learning context. The reason might be that team training offers the opportunity to work as a workforce that can deliver compassion as a strategic and synergic process [8–12]. Moreover, the training could be an opportunity to stimulate reflections and considerations among a multidisciplinary team.

Sinclair et al.’s [3] grounded theory model, the Healthcare Compassion Model, could be an adequate starting point to implement specific teaching and training programs for palliative care professionals. We do not know in detail how compassion is addressed in the current curriculum for medical education or medical training in Italy. However, in Italy, law Number 38 of 2010, that for the first time guarantees the citizen’s right to access palliative care and pain therapy, does not provide for any training on compassion [13]. Furthermore, compassion and training about this theme are not expected in the core curriculum of HCPs in palliative care [14].

While, according to the results of the survey, only 11 healthcare professionals received compassion training during academical studies. Thus, data suggest that participants’ training occasions on compassion have been scarce, and, at the same time, they seem to be necessary and useful.

## Limitations and Insight for Future Research

The research has several limitations.

The snowball sampling did not allow managing recruitment, opening to possible selection biases or preventing variability on factors of interest.

Moreover, most participants were 50–60 years old; thus, it would be useful to do a study on young HCPs to understand whether training opportunities on compassion have increased.

Then, the results of the study are not generalizable to other countries because of cultural and educational differences.

Finally, the present study is a simple opinion survey: more detailed results could be obtained using more demographic variables of interest. Further research with a larger sample might include a specific evaluation of efficacy of compassion training intervention.

## Conclusions

Compassion is considered an essential construct both in clinical practice and in palliative care international guidelines. These findings might indicate the increasing importance of offering team training and sharing occasions that can promote a deeper awareness of the construct and its utility in clinical practice.

## References

[1] Larkin P (2015) Compassion: the essence of palliative and end-of-life care. Oxford University Press

[2] Sinclair S, McClement S, Raffin-Bouchal S, Chochinov HM, Hagen NA (2016). Compassion in health care: an empirical model. J Pain Symptom Manage.

[3] Brito-Pons G, Librada-Flores S (2018). Compassion in palliative care: a review. Curr Opin Support Palliat Care.

[4] Sinclair S, Beamer K, Hack TF, McClement S, Raffin-Bouchal S, Chochinov HM, Hagen NA (2017). Sympathy, empathy, and compassion: a grounded theory study of palliative care patients’ understandings, experiences, and preferences. Palliat Med.

[5] Sinclair S, Torres M-B, Raffin-Bouchal S, Hack TF, McClement S, Hagen NA, Chochinov HM (2016). Compassion training in healthcare: what are patients’ perspectives on training healthcare providers?. BMC Med Educ.

[6] Scarlet J, Altmeyer N, Knier S, Harpin RE (2017). The effects of Compassion Cultivation Training (CCT) on health-care workers. Clin Psychol.

[7] Sinclair S, Hack TF, Raffin-Bouchal S, McClement S, Stajduhar K, Singh P, Hagen NA, Sinnarajah A, Chochinov HM (2018). What are healthcare providers’ understandings and experiences of compassion? The healthcare compassion model: a grounded theory study of healthcare providers in Canada. BMJ Open.

[8] Contratti F, Ng G, Deeb J (2012). Interdisciplinary team training: five lessons learned. Am J Nurs.

[9] Bridges J, Fuller A (2015). Creating learning environments for compassionate care: a program to promote compassionate care by health and social care teams. Int J Older People Nurs.

[10] de Geer V, Joep NV, Groot M, Zock H, Prins CLJ, Vissers K (2018). Multidisciplinary training on spiritual care for patients in palliative care trajectories improves the attitudes and competencies of hospital medical staff: results of a quasi-experimental study. Am J Hosp Palliat Med.

[11] Baldwin PK, Wittenberg-Lyles E, Oliver DP, Demiris G (2011). An evaluation of interdisciplinary team training in hospice care. J Hosp Palliat Nurs.

[12] Goldsmith J, Wittenberg-Lyles E, Rodriguez D, Sanchez-Reilly S (2010). Interdisciplinary geriatric and palliative care team narratives: collaboration practices and barriers. Qual Health Res.

[13] Legge 15 Marzo 2010, n. 38. Disposizioni per garantire l'accesso alle cure palliative e alla terapia del dolore. (10G0056), G.U. n. 65 del 19 marzo 2010. Retrieved on https://www.parlamento.it/parlam/leggi/10038l.htm. Accessed 21 June 2021

[14] Società Italiana di Cure Palliative (SICP). Core curriculum. https://www.sicp.it/aggiornamento/core-curriculum/. Accessed 21 June 2021

